# Impact of CT-measured sarcopenic obesity on postoperative outcomes following colon cancer surgery

**DOI:** 10.1007/s00423-024-03231-0

**Published:** 2024-01-17

**Authors:** Mariam Bajawi, Sara Corral, Javier Blázquez, Javier Die, Paula Muñoz, Alberto G. Barranquero, Luz Juez, Francisca García-Moreno Nisa

**Affiliations:** 1 University of Alcalá, Alcalá de Henares, Spain; 2 Department of General Surgery, Ramón y Cajal University Hospital, Madrid, Spain; 3 Department of Radiology, Ramón y Cajal University Hospital, Madrid, Spain

**Keywords:** Sarcopenic obesity, Anastomotic leak, Colon cancer, Colon cancer surgery

## Abstract

**Objective:**

This study aimed to investigate the influence of sarcopenic obesity on anastomotic leak following elective colon resection for non-metastatic colon cancer. Secondary outcomes included overall morbidity, mortality and length of hospital stay.

**Methods:**

This retrospective observational study, conducted at a colorectal surgery referral centre, spanned from January 1, 2015, to January 1, 2020. A total of 544 consecutive patients who underwent elective colon resection were included in the analysis, excluding patients with rectal cancer, urgent surgery, absence of anastomosis, lack of imaging, multivisceral resections and synchronic tumours.

**Results:**

Postoperative complications were observed in 177 (32.3%) patients, with 51 (9.31%) classified as severe (Clavien-Dindo > II). Sarcopenic obesity was identified in 9.39% of the sample and emerged as an independent predictor of increased overall morbidity [OR 2.15 (1.14–3.69); *p* = 0.016] and 30-day mortality [OR 5.07 (1.22–20.93); *p* = 0.03] and was significantly associated with the development of anastomotic leak [OR 2.95 (1.41–6.18); *p* = 0.007]. Furthermore, it increased the risk of reoperation and was linked to a prolonged length of hospital stay.

**Conclusions:**

CT-measured sarcopenic obesity demonstrates a discernible correlation with an elevated risk of postoperative morbidity and mortality in the context of colon cancer surgery.

## Introduction

Sarcopenic obesity (SO) is currently defined as decreased muscle mass and function in obese individuals [1]. The pathogenesis of SO is marked by a bidirectional association between sarcopenia and obesity. On the one hand, diminished skeletal muscle mass can lead to a reduction in resting and total metabolic output, thereby promoting fat accumulation. On the other hand, obesity may facilitate the development and progression of sarcopenia. This occurs through a multifactorial network of abnormalities, primarily involving the proinflammatory state produced by visceral adipose tissue [2]. Independently, both sarcopenia and obesity pose an increased risk of adverse health outcomes. When these conditions coexist, the risks are synergistically amplified.

Computed tomography (CT) stands as the “gold standard” for non-invasive assessment of muscle quantity and quality, benefitting from its exceptional reproducibility [3, 4]. Moreover, CT is routinely incorporated into the preoperative evaluation for the majority of colon cancer patients.

SO is associated with an elevated incidence of postoperative complications, prolonged hospital stays, and heightened hospitalisation costs [3–6]. This study aims to examine the correlation between preoperative CT measures of SO and short-term surgical outcomes within a relatively homogeneous group of non-metastatic colon cancer patients. Our hypothesis proposes that radiologic assessment of SO can serve as a predictive indicator for adverse short-term outcomes.

## Materials and methods

A retrospective observational study was conducted on 544 patients who underwent elective colon resection (including left and right, simple and extended, hemicolectomy, and sigmoidectomy) for non-metastatic colon cancer at a tertiary-level hospital in Madrid, Spain, between January 2015 and January 2020. The sample size was guided by the practicality of accessing available data and conducting a thorough investigation into our research objectives. Factors taken into account encompass the representativeness of the sample, the accessibility of data, and the statistical power required for detecting significant associations.

The study adhered to ethical standards for retrospective research, with approval granted by the Ethics Committee of the Ramón y Cajal University Hospital (No. 374/20) to utilise information obtained from medical records.

**Fig. 1:**
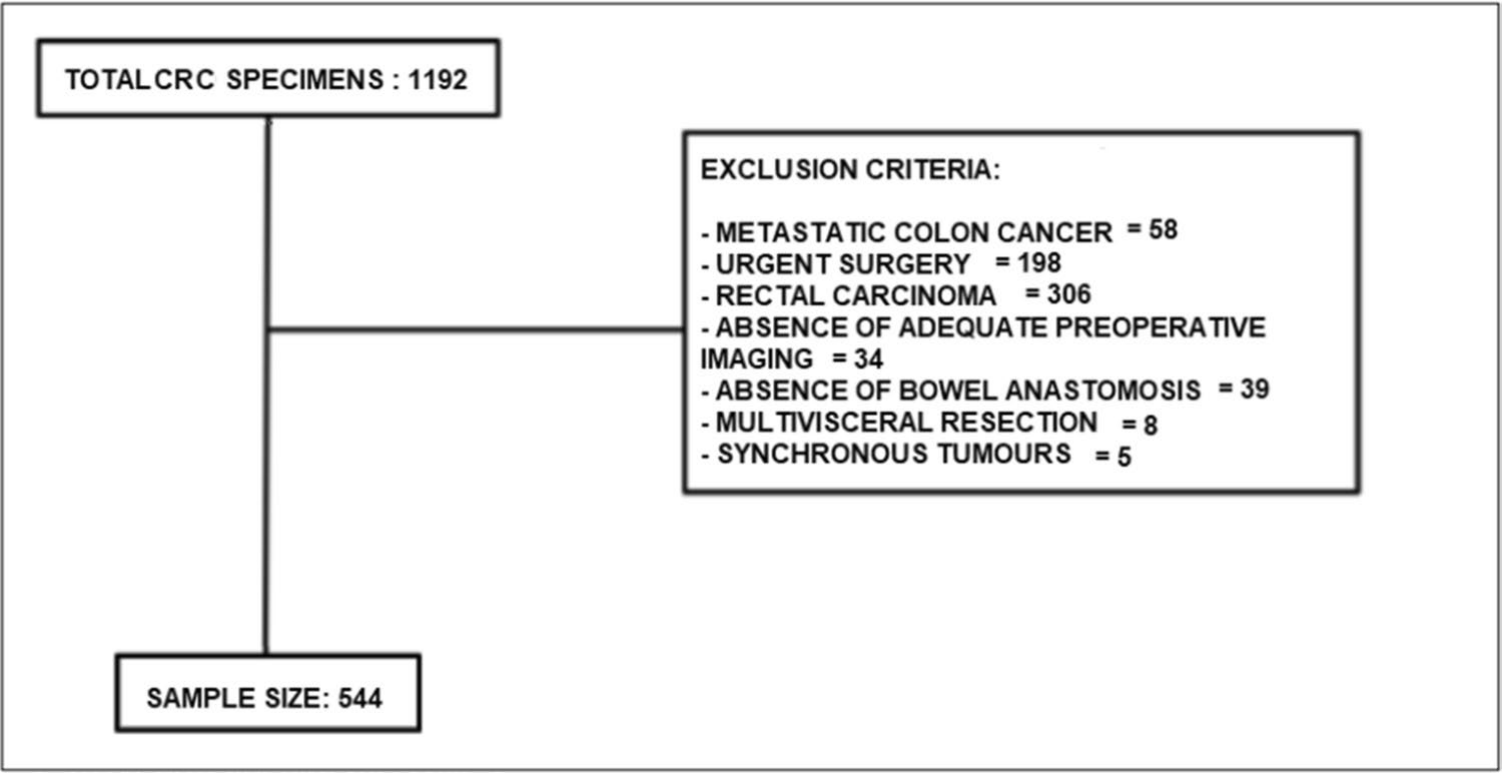
Flowchart of the study sample, depicting exclusion criteria and sample size. CRC: colorectal cancer

All surgical specimens with colorectal cancer results from the specified period were retrieved, totalling 1192 samples. After applying exclusion criteria, the final sample size was obtained (see Fig. 1).

### Short-term postsurgery morbidity

The Clavien-Dindo complication grading system was employed to categorise documented outcomes before conducting statistical analysis. Patients experiencing multiple complications were classified based on the highest grade among the reported complications [7, 8].

### Body composition parameter measurements

Radiological measurements were made using diagnostic imaging software Synapse’s PACS® (picture archiving and communication system), FUJIFILM Medical Systems USA, Inc. Morrisville, North Carolina. Imaging modality used was computerised tomography scan, and the analysis was obtained from two-dimensional (2D) and three-dimensional (3D) images. Image slice thickness was of 3 mm without gap, low noise and axial cuts. Anatomical region included the upper border of the liver to the upper border of the pubic symphysis in supine position. The diagnosis was established in the presurgical CT scan at the level of the third lumbar vertebra (L3).

Imaging was based on automatic detection of the psoas muscle and calculation of its area and manual detection of paraspinal and abdominal wall muscles (transversus abdominis, external and internal obliques, rectus abdominis) and calculation of their area. To identify muscle tissue, the range of − 29 to + 150 in Hounsfield Units (HU) was used. The skeletal muscle mass index was calculated as the sum of the cross-sectional areas of these muscles (cm^2^).

The radiological diagnosis of obesity was established based on the semiautomatic detection and frequency distribution of subcutaneous and visceral fat in one plane (2D) at the L3 level. To identify fatty tissue, the range between − 200 and − 50 in Hounsfield Units (HU) was used. The body fat index was calculated with the sum of the cross-sectional areas (cm^2^).

Subsequently, all body composition measurements were standardised using height in metres squared and expressed as cm^2^/m^2^, according to Mosteller’s formula. Total skeletal muscle area (TSMA), subcutaneous fat area (SFA) and visceral fat area (VFA) were used to calculate the total skeletal muscle index (TSMI = TSMA/m^2^), the subcutaneous adipose tissue index (SATI = SFA/m^2^) and the visceral adipose tissue index (VATI = VFA/m^2^), respectively. Finally, we evaluated the relationship between VFA and TSMA, that is, the visceral fat muscle area index (VFMAI = VFA/TSMA), which was used as a measurement of SO.

For the numerical definition of sarcopenia, the values of Prado et al. and their subsequent modification according to BMI published by Martin et al. were used. According to them, for males with BMI < 25, a TSMI < 43 cm^2^/m^2^ was used; for males with BMI > 25, a TSMI < 53 cm^2^/m^2^ was used; and for females, a TSMI < 41 cm^2^/m^2^ was selected [9].

In determining the obesity cut-off values, we adhered to the criteria outlined by Juez et al. Specifically, for males, the identified cut-off values for VFA and SFA were 153.8 and 128.6, respectively. Conversely, for females, the corresponding cut-off values were 121.05 for VFA and 163.17 for SFA [10]. VFA was selected as the representative measure of body fat due to its superior sensitivity and specificity. Furthermore, it aligns with international trends where the majority of publications favour this parameter as the designated cut-off point for defining obesity.

### Statistical analysis

The normality of variables was assessed using the Shapiro–Wilk test. Categorical data were delineated through absolute frequencies and percentages, whereas numerical data were conveyed using means with standard deviations, medians, and ranges (minimum and maximum values). Comparison between qualitative data utilised the chi-square test or Fisher’s exact test, and for quantitative data, Student’s *t*-test or the Mann–Whitney *U* test for ordinal data was employed.

Univariate and multivariate analyses with logistic regression were conducted to identify factors associated with a higher rate of postoperative complications in colon cancer surgery. The degree of association was estimated using corresponding odds ratios (OR) and their 95% confidence intervals. Statistical significance was considered at a *p* value < 0.05.

For variables that showed statistical significance in the multivariate analysis, a ROC curve analysis was performed, and the area under the curve (AUC) was calculated. To assess diagnostic reliability, the Swets classification [11] was applied, categorising each diagnostic test based on the AUC value.

The analysis was carried out using STATA® Statistics software (Version 16), developed by StataCorp, USA, College Station, Texas.

## Results

Clinical and pathological characteristics of 544 patients included in the final analysis were stratified by sex, as presented in Table 1. Sarcopenic obesity was identified in 45 patients, comprising 14.4% of males and 6.4% of females.

**Table 1:** Characteristics of the sample

	Males (*n*, 310)	Females (*n*, 234)
Mean age, years (± SD)	71.9 ± 11	73.5 ± 11
BMI Underweight Normal Overweight Obese	1.94% 24.52% 50.97% 22.58%	4.7% 34.62% 36.32% 24.36%
ASA I II III IV	4.70% 58.97% 35.47% 0.85%	5.81% 55.16% 38.39% 0.65%
HTN	62.46%	58.55%
DM	26.77%	17.52%
Hypercholesterolemia	32.36%	35.04%
Heart disease	20.71%	14.53%
Cerebrovascular disease	8.77%	8.15%
PAD	6.13%	0.43%
Smoking Active Ex-smoker	15.81% 30.65%	11.54% 8.55%
Chronic lung disease	13.92%	11.54%
CKD	11.61%	4.70%
Dementia	2.58%	2.56%
Chronic liver disease	5.16%	3.85%
Anaemia	57%	42%
Hypoproteinaemia	24.2%	26.5%
Visceral obesity	68.1%	50.7%
Sarcopenia	19.8%	14.3%
Sarcopenic obesity	14.4%	6.2%
Previous abdominal surgery	23.6%	32.5%
Tumour location Right-sided Left-sided	42.2% 57.8%	57.5% 42.5%
TNM I II III	24.84% 40% 35.15%	29.91% 37.17% 32.91%
Approach Laparoscopic Open Conversion	54.70% 38.46% 7.10%	30.32% 62.58% 6.84%

Complications, classified according to the Clavien-Dindo system, were observed in 177 patients (32.3%), with 51 cases (9.31%) classified as severe (Clavien-Dindo > II). The overall Clavien-Dindo distribution in the sample revealed varying levels of postoperative complications. Class I comprised 27.07% (*n* = 49), Class II made up 44.75% (*n* = 81), Class IIIa was at 4.42% (*n* = 8), Class IIIb was 14.92% (*n* = 27), Class IVa accounted for 2.21% (*n* = 4), and Class IVb represented 4.97% (*n* = 9).This study specifically investigated four predominant abdominal postoperative adverse events: anastomotic leak, paralytic ileus, surgical site infection (SSI), and haemorrhagic complications. Anastomotic leak emerged as the most prevalent complication, affecting 53 patients (9.74%), followed by paralytic ileus (9.01%), SSI occurring in 8.46% of the sample, and, notably, 27 cases (5.06%) of postoperative bleeding.

The univariate and multivariate analyses, detailed in Table 2, identified male sex [OR 1.79 (1.22–2.64); *p* = 0.003], hypercholesterolaemia [OR 1.90 (1.29–2.81); *p* = 0.001], hypoproteinaemia [OR 1.59 (1.04–2.42); *p* = 0.031], and sarcopenic obesity [OR 2.15 (1.14–3.69); *p* = 0.016] as independent risk factors for overall postoperative morbidity.

**Table 2:** Univariate and multivariate analyses for postoperative morbidity risk factors

	Postoperative morbidity *n* = 177	No postoperative morbidity *n* = 366	Univariate analysis *p* value	Multivariate analysis	
				Odds ratio (95% CI)	*p* value
Mean age, years (± SD)	74.6 ∓ 9.9	71.6 ∓ 11.1	**0.002**		
Sex Male Female	114 (36.77%) 63 (27.04%)	196 (63.23%) 170 (72.96%)	**0.017**	1.22–2.64	0.003
BMI < 30 > = 30	140 (33.57%) 37 (29.37%)	277 (66.43%) 89 (70.63%)	0.377		
ASA < III = > III	93 (27.60%) 84 (40.78%)	244 (72.40%) 122 (59.22%)	**0.001**		
HTN	118 (66.67%)	211 (57.81%)	**0.048**		
DM	77 (21.04%)	47 (26.55%)	0.151		
Hypercholesterolaemia	47 (26.55%)	77 (21.04%)	**0.001**	1.29–2.81	0.001
Heart disease	64 (17.53%)	34 (19.21%)	0.635		
Cerebrovascular disease	16 (9.14%)	30 (8.22%)	0.719		
PAD	11 (6.21%)	9 (2.46%)	**0.029**		
Smoking Active Ex-smoker	20 (11.30%) 45 (25.42%)	56 (15.30%) 70 (19.13%)	0.158		
Chronic lung disease	28 (15.82%)	42 (11.51%)	0.160		
CKD	23 (12.99%)	24 (6.56%)	**0.012**		
Dementia	5 (2.82%)	9 (2.46%)	0.801		
Chronic liver disease	10 (5.65%)	15 (4.10%)	0.421		
Anaemia	53 (29.94%)	67 (18.31%)	**0.002**		
Hypoproteinaemia	53 (29.94%)	67 (18.31%)	**0.002**	1.04–2.42	0.031
Visceral obesity	122 (68.93%)	223 (61.74%)	0.051		
Sarcopenia	32 (18.08%)	59 (16.12%)	0.567		
Sarcopenic obesity	24 (13.56%)	26 (7.10%)	**0.015**	1.14–3.69	0.016
Previous abdominal surgery	56 (31.64%)	92 (25.14%)	0.111		
Tumour location Right-sided Left-sided	103 (35.3%) 74 (29.5%)	189 (64.7%) 177 (70.5%)	0.151		
TNM I II III	47 (32%) 70 (33%) 60 (32%)	100 (68%) 140 (67%) 126 (68%)	0.957		
Approach Laparoscopic Open Conversion	92 (51.98%) 73 (41.24%) 12 (6.78%)	192 (52.46%) 148 (40.44%) 26 (7.10%)	0.979		

The values that are emphasized through bold or underlining are indicative of statistical significance within the context of this analysis

Table 3 illustrates that postoperative mortality is independently associated with older age [OR 1.14 (1.02–1.27); *p* = 0.020], dementia [OR 12.85 (2.12–77.83); *p* = 0.005], chronic liver disease [OR 17.36 (3.49–86.31); *p* = 0.000], and sarcopenic obesity [OR 5.07 (1.22–20.93); *p* = 0.03]. Our mortality prediction model was constructed using forward stepwise logistic regression, yielding these four factors as outcomes, with an area under the curve of 0.9171.

**Table 3:** Univariate and multivariate analyses for postoperative mortality risk factors

	Postoperative mortality *n* = 9 (1.65%)		Univariate analysis *p* value	Multivariate analysis	
				Odds ratio (95% CI)	*p* value
Mean age, years (± SD)	82 ± 6.17	72 ± 10.83	**0.008**	1.02–1.27	0.02
Sex Male Female	6 (1.94%) 3 (1.28%)	304 (98.06%) 231 (98.72%)	0.557		
BMI < 30 > = 30	7 (1.68%) 2 (1.57%)	410 (98.32%) 125 (98.43%)	0.936		
ASA < III = > III	3 (0.89%) 6 (2.91%)	335 (99.11%) 200 (97.09%)	0.090		
HTN	8 (2.42%)	1 (0.47%)	0.082		
DM	5 (4.03%)	4 (0.95%)	**0.018**		
Hypercholesterolaemia	6 (3.30%)	3 (0.83%)	**0.034**		
Heart disease	4 (4.08%)	5 (1.12%)	**0.038**		
Cerebrovascular disease	0	9 (1.82%)			
PAD	1 (5.00%)	8 (1.53%)	0.232		
Smoking Active Ex-smoker	0 3 (33.33%)	76 (14.21%) 112 (20.93%)	0.541		
Chronic lung disease	0	70 (13.11%)			
CKD	2 (4.26%)	7 (1.41%)			
Dementia	2 (14.29%)	7 (1.32%)	**0.003**	2.12–77.83	0.005
Chronic liver disease	3 (12.00%)	6 (1.16%)	**0.001**	3.49–86.31	0.000
Anaemia	4 (3.33%)	5 (1.18%)	0.102		
Hypoproteinaemia	4 (2.92%)	5 (1.23%)	0.179		
Visceral obesity	7 (77.78%)	324 (60.56%)	0.294		
Sarcopenia	92 (17.20%)	3 (33%)	0.206		
Sarcopenic obesity	48 (8.97%)	3 (33%)	**0.013**		
Previous abdominal surgery	4 (2.68%)	5 (1.27%)	0.247		
Tumour location Right-sided Left-sided	6 (2.05%) 3 (1.2%)	286 (97.95%) 249 (98.8%)	0.431		
TNM					
I II III	3 (2.04%) 4 (1.9%) 2 (1.08%)	144 (97.96%) 207 (98.10%) 184 (98.92%)	0.743		
Approach					
Laparoscopic Open Conversion	6 (2.11%) 3 (1.35%) 0	278 (97.89%) 219 (98.65%) 38 (100%)	0.568		

The values that are emphasized through bold or underlining are indicative of statistical significance within the context of this analysis

Regarding specific abdominal complications (Table 4), sarcopenic obesity increased the risk of anastomotic leak [OR 2.95 (1.41–6.18); *p* = 0.007], while it exhibited no significant influence on the incidence of paralytic ileus (*p* = 0.94), SSI (*p* = 0.91), or postoperative bleeding (*p* = 0.765). Patients with sarcopenic obesity experienced prolonged hospital stays [OR 1.75 (1.18–2.60); *p* = 0.006] and an increased risk of reoperation [OR 1.48 (1.01–2.17); *p* = 0.047]. However, no effects on readmission (*p* = 0.54) or operative time (*p* = 0.08) were demonstrated.

**Table 4:** Effect of sarcopenic obesity on colon cancer surgery and postoperative outcomes

	*p* value	OR	95% CI
Overall morbidity	**0.009**	0.041	1.02–2.35
Post-op mortality	**0.003**	0.007	1.77–36.34
Anastomotic leak	0.218	0.024	1.05–1.92
Postoperative bleeding	0.765	1.09	0.62–1.91
Ileo paralítico	0.94	1.02	0.65–1.60
Paralytic ileus	0.94	1.02	0.65–1.60
Abdominal wound dehiscence	0.69	0.73	0.17–3.08
Surgical site infection	0.91	0.73	0.53–1.56
Readmission	0.54	1.23	0.63–2.38
Reoperation	**0.047**	1.48	1.01–2.17
Operative time	0.08	0.77	− 10.18–7.58
Length of hospital stay			

The values that are emphasized through bold or underlining are indicative of statistical significance within the context of this analysis

## Discussion

The current study investigates the intricate relationship between sarcopenic obesity (SO) and postoperative complications in colon cancer surgery. The findings suggest that SO could serve as a significant risk factor for increased morbidity and mortality, particularly impacting anastomotic leak rates. Interestingly, the study challenges the conventional focus on body mass index (BMI) as the sole indicator of obesity, emphasising the need for a more nuanced understanding of obesity that includes factors like visceral fat distribution.

While the study provides valuable insights, it is essential to acknowledge its limitations. The failure to demonstrate significant differences in complications between obese and non-obese patients, as seen in other cohorts, raises questions about the generalizability of the findings. Additionally, the study acknowledges the lack of consensus on cut-off points for defining radiological sarcopenia, potentially influencing the assessment of its predictive value in postoperative complications.

Despite its limitations, the study contributes to the existing body of knowledge by proposing a novel perspective on the interplay between SO and postoperative outcomes. The incorporation of a comprehensive model for predicting postoperative morbidity, including factors like sex, hypercholesterolemia, hypoproteinaemia, and SO, demonstrates a thorough analytical approach. The study’s strength lies in its ability to prompt a reevaluation of conventional obesity measures and advocate for a more nuanced understanding of the factors contributing to postoperative complications.

Our sample exhibited a 9.34% incidence, consistent with results reported in the literature, which range from 6 to 18%[2, 6]. Notably, our analysis failed to demonstrate an independent association between sarcopenia, visceral obesity, and postoperative abdominal complications. However, when combined into the form of SO, the risk increased twofold, suggesting that SO might be a more accurate indicator of morbidity.

Similar findings in other studies support the link between SO and postoperative complications [3, 12–17]. For instance, Chen et al. [3] reported a surgical complication rate of 6.7% in the non-SO group compared to 31.7% in the SO group (*p* < 0.001). Pedrazzani et al. [13], in a study of 261 patients undergoing laparoscopic resection for colorectal cancer, found SO to be a risk factor for medical and surgical complications. Malietzis et al. [4] identified a higher risk of major complications (22 vs. 13.0%; *p* = 0.019) in patients with SO.

In the context of anastomotic leak, preoperative identification of SO proved valuable in predicting gastric leaks after sleeve gastrectomy [18] and increased the risk of pancreatic fistula after pancreaticoduodenectomy [19]. Additionally, SO was associated with severe postoperative complications and poorer long-term survival after gastrectomy for gastric cancer [10]. In colon cancer surgery, our study observed an almost threefold increase in the rate of anastomosis dehiscence in the SO group.

Concerning postoperative mortality, detailed preoperative clinical data allowed us to assess risk factors for mortality, revealing SO as a relatively new but potent predictor in colon cancer surgery. The association between SO and immediate postoperative mortality was evident [5.07 (1.22–20.93); *p* = 0.03], aligning with its link to abdominal complications and, specifically, anastomotic leak, the latter being a leading cause of postoperative mortality in our cohort.

The findings highlight the critical role of preoperative patient condition in postoperative complications, emphasising the importance of preoperative rehabilitation, consistent with previous studies on risk factors for postoperative complications.

The model’s clinical utility, population specificity, and robust predictive capacity simplify and expedite the consent process, providing an additional tool in presurgical rehabilitation protocols. This is crucial as patients undergoing surgical prehabilitation experience benefits such as improved physiology during surgery, reduced complication rates, and lower costs [20]. While studies on short-term preoperative prehabilitation for sarcopenia exist, research demonstrating tools to improve preoperative SO and their impact on postoperative outcomes is lacking [21, 22].

CT-based measurement of SO proves an easily implementable tool, incurring no significant monetary or physical/psychological burden. Its integration into the staging of colon tumours, with feasible calculations, adds to its practical appeal.

Beyond immediate applications, the study urges exploration into the impact of CT-measured sarcopenic obesity on overall survival postcolon cancer surgery. Assessing potential disparities in overall survival rates offers a comprehensive perspective, crucial for refining risk stratification models and tailoring interventions. This endeavour aligns with evolving strategies for nuanced prognostication and personalised treatment plans, enhancing our understanding of the enduring effects of sarcopenic obesity on colorectal surgery outcomes and overall patient survival.

Apart from the clinical applications highlighted in this study, a promising avenue emerges for future research that could impact preoperative assessment methodologies. The accessibility and non-invasiveness of ultrasound imaging present an intriguing opportunity to explore its utility in measuring sarcopenia, a key component of sarcopenic obesity. An exciting prospect is the validation of ultrasound as a reliable tool for measuring sarcopenia. While computed tomography is commonly employed for this purpose, our study could serve as a stepping stone for future investigations focusing on validating ultrasound measurements against established CT-based criteria for sarcopenia, providing a more practical approach to preoperative assessment.

## Conclusions

In conclusion, our study highlights the significant association between CT-measured SO and elevated risks of postoperative morbidity and mortality in the context of colon cancer surgery. Particularly, the increased likelihood of anastomotic leak underscores the clinical relevance of identifying and addressing SO in preoperative assessments. The study suggests that CT measurements of SO could serve as a practical and easily implementable tool in surgical prehabilitation programs.

However, it is essential to acknowledge that our findings necessitate further validation through prospective clinical trials. These trials are crucial for determining the most effective measures to improve preoperative SO, especially considering the time-sensitive nature of colon cancer surgery. Continued research in this direction will not only refine risk assessment models but also contribute to the development of targeted interventions, optimising patient outcomes in the challenging context of colon cancer surgery.
